# Identification of galangin as a therapeutic candidate for primary biliary cholangitis via systematic druggable genome-wide Mendelian randomization analysis and experimental validation

**DOI:** 10.3389/fphar.2025.1674693

**Published:** 2025-10-09

**Authors:** Weirui Ren, Chuang Zhang, Hanyan Wang, Hongzhao Song, Xuejuan Zhu, Zhijun Zhang, Suxian Zhao, Junmin Wang

**Affiliations:** ^1^ Department of Gastroenterology, Hebei Medical University Third Hospital, Shijiazhuang, China; ^2^ Department of Clinical Medicine, Shijiazhuang Medical College, Shijiazhuang, China; ^3^ Pharmacy Intravenous Admixture Services, Hebei Medical University Third Hospital, Shijiazhuang, China; ^4^ Department of Graduate College, Hebei Medical University, Shijiazhuang, China; ^5^ Department of Traditional and Western Medical Hepatology, Hebei Medical University Third Hospital, The Key Laboratory of Hepatic Fibrosis Mechanisms of Chronic Liver Diseases in Hebei Province, Hebei International Science and Technology Cooperation Base – Hebei International Joint Research Center for Molecular Diagnosis of Liver Cancer, Shijiazhuang, China

**Keywords:** primary biliary cholangitis, Mendelian randomization, druggable genome, galangin, ADORA2A

## Abstract

**Background:**

Primary biliary cholangitis (PBC) is an immune-mediated cholestatic liver disease with currently limited therapeutic options. This study aimed to identify novel therapeutic targets for PBC via systematic druggable genome-wide Mendelian randomization (MR) analysis, predict candidate drugs, and experimentally validate the candidates.

**Methods:**

The study integrated druggable genome data, cis-expression quantitative trait loci (cis-eQTL) in blood and liver tissues, and summary data from PBC genome-wide association studies (GWAS). Two-sample MR analysis and colocalization analysis were used to screen genes significantly associated with PBC, followed by phenome-wide association study (PheWAS), functional enrichment analysis, protein-protein interaction (PPI) network construction, drug prediction, and molecular docking. Finally, the therapeutic potential of the candidate drug galangin (GAL) was validated using an α-naphthylisothiocyanate (ANIT)-induced PBC mouse model.

**Results:**

A total of 15 druggable genes significantly associated with PBC were identified, primarily enriched in biological processes regulating immune homeostasis, inflammatory signaling, and apoptosis, among others. Subsequent bioinformatic drug prediction and molecular docking identified GAL as a promising drug candidate, showing strong binding affinity to the target ADORA2A. Animal experiments showed that GAL reduced portal tract inflammation and bile duct hyperplasia in liver tissues, while reducing serum levels of liver enzymes (ALT, AST, ALP, etc.) and hepatic expression of inflammatory cytokines (IL-1β, IL-6, TNF-α).

**Conclusion:**

By integrating systematic druggable genome-wide MR analysis with experimental validation, this study identified 15 druggable genes associated with PBC. More importantly, it identified GAL as a therapeutic candidate for PBC, with its effects potentially mediated by the ADORA2A target. These findings provide novel therapeutic targets and drugs for PBC. Future research will focus on validating the functions of these druggable genes and elucidating the mechanistic pathway of the galangin-ADORA2A interaction, laying a more solid and comprehensive theoretical and practical foundation for PBC treatment.

## Highlights

• Via systematic druggable genome-wide MR analysis, 15 PBC-associated druggable genes were identified, which are enriched in biological processes regulating immune homeostasis, inflammatory signaling, and apoptosis, among others.
• Bioinformatic drug prediction and molecular docking identified GAL as a promising drug candidate, showing strong binding affinity to the target ADORA2A.
• Animal experiments demonstrated that GAL reduced portal tract inflammation and bile duct hyperplasia in liver tissues while lowering serum liver enzymes and hepatic inflammatory cytokines, further supporting its potential as a therapeutic candidate for PBC.

## 1 Introduction

Primary biliary cholangitis (PBC) is a common immune-mediated cholestatic liver disease (CLD), primarily affecting middle-aged to elderly females. It causes chronic inflammation and damage to intrahepatic bile ducts, which may progress to hepatic fibrosis and liver failure (Patel et al., 2021; Geng et al., 2025; Selmi et al., 2011). The pathogenesis of PBC is complex, involving interactions among genetic, environmental, and immune factors. These factors together activate the immune system, trigger chronic inflammatory responses, destroy bile duct epithelial cells, and eventually lead to cholestasis and liver injury (Jones, 2003; Gershwin et al., 2005; Wang et al., 2023). In PBC treatment, existing pharmacological options remain limited. Ursodeoxycholic acid (UDCA) serves as the first-line therapy, but approximately 40% of patients show inadequate response. Obeticholic acid has demonstrated efficacy in UDCA non-responders as a second-line treatment. However, it is contraindicated in cirrhotic patients with portal hypertension, and many patients experience intolerable pruritus (Barba Bernal et al., 2023; Xia et al., 2022). The Phase 3 trial results of Elafibranor and Seladelpar, published in 2024, represent a significant breakthrough in PBC treatment. These agents markedly improve liver enzyme levels and alleviate itching, providing new hope for patients with inadequate responses to UDCA. However, challenges remain, including insufficient long-term data, suboptimal responses in some patients, and unresolved side effects (Hirschfield et al., 2024; Kowdley et al., 2024). Therefore, exploring novel therapeutic strategies is of great significance, and the continuous exploration of potential therapeutic targets for PBC has become a research focus. Integrating genetics into drug development has provided new ideas for this field. In particular, the proposal of the concept of “druggable genome” has significantly improved the efficiency of drug discovery (Zhang et al., 2024; Yin et al., 2024).

Genome-wide association studies (GWAS) are effective in identifying single nucleotide polymorphisms (SNPs) associated with PBC. However, due to linkage disequilibrium, the local correlation of multiple genetic variants can only be preliminarily identified at a single locus, which hinders the identification of causal variants (Kar et al., 2013; Gerussi et al., 2022). Moreover, without comprehensive downstream analysis, this approach struggles to directly locate pathogenic genes or offer clear directions for drug development (Zhang et al., 2024). Consequently, integrating GWAS genetic information with other biological mechanism studies has become an effective means to promote the discovery of novel therapeutic targets. Mendelian randomization (MR) integrates disease GWAS data with expression quantitative trait loci (eQTL) data, using genetic variations as instrumental variables (IVs) to infer causal relationships between exposures and disease outcomes, thereby significantly enhancing the reliability of causal inference (Lovegrove et al., 2024). Currently, the research strategy integrating the druggable genome with cis-expression quantitative trait loci (cis-eQTL) MR analysis has been widely adopted. This approach efficiently identifies novel therapeutic targets and has successfully determined potential treatment targets for various complex diseases (Zhang et al., 2024; Yin et al., 2024; Su et al., 2023).

Building on the advantages of integrated research methods, this study aimed to identify novel therapeutic targets for PBC through systematic druggable genome-wide Mendelian randomization (MR) analysis, conduct candidate drug prediction, and experimentally validate the candidate drugs. To this end, we integrated druggable genome data, screened genes within blood and liver cis-eQTLs, and performed two-sample MR analysis by integrating these genes with PBC GWAS data to identify genes significantly associated with PBC. We then conducted colocalization analysis to ensure the robustness of results, followed by phenome-wide association studies (PheWAS), enrichment analysis, construction of a protein-protein interaction (PPI) network, drug prediction, and molecular docking. Finally, we evaluated the therapeutic effects of candidate drug interventions using an established animal model *in vivo*. This study provides novel therapeutic targets and promising candidate drugs for PBC treatment, laying a more solid and comprehensive theoretical and practical foundation for PBC treatment.

## 2 Materials and methods

### 2.1 Research design

**FIGURE 1:**
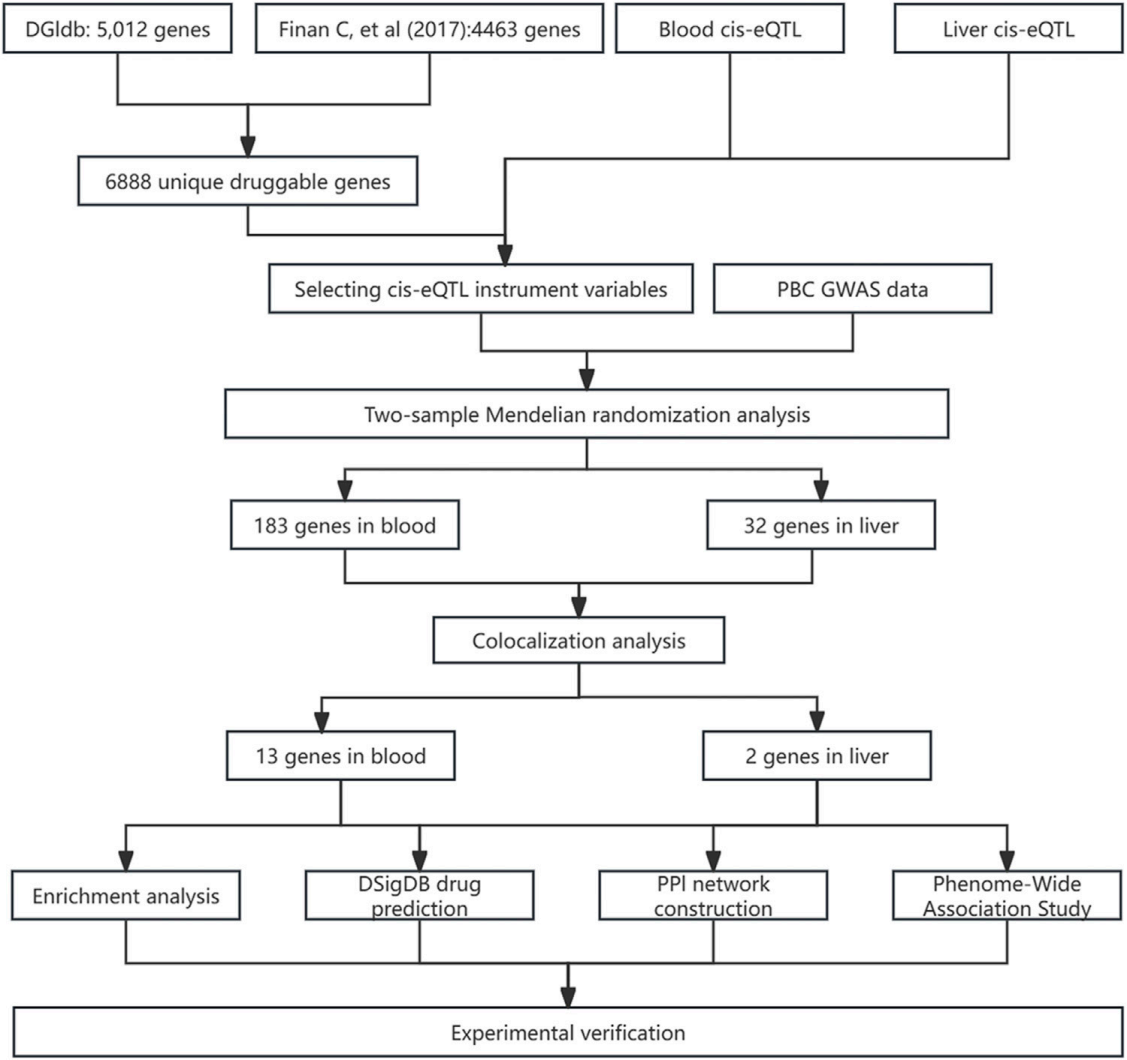
Flowchart of the analysis process in this article.

Figure 1 outlines the study’s flow chart. First, druggable genes were retrieved from the Drug-Gene Interaction Database (DGIdb, https://www.dgidb.org/) and a review article. Cis-eQTL data for blood and liver tissues were then sourced from eQTLGen and GTEx V10, respectively. Using these data, we identified cis-eQTL IVs and performed two-sample MR analysis with PBC GWAS data to screen for disease-associated genes. Colocalization analysis validated the robustness of these associations. Subsequently, PheWAS (FinnGen v11) assessed the safety profiles of colocalized genes as drug targets. Functional analyses and PPI network construction explored the biological roles of candidate proteins. Drug prediction and molecular docking were performed on prioritized genes. Finally, we integrated the above results with existing evidence to evaluate potential therapies and experimentally validated therapeutic candidates in a mouse model of PBC.

### 2.2 Druggable genes

Given that not all genes are suitable for direct drug development, we identified drug target genes using DGIdb and a review by Finan et al. (Finan et al., 2017). These genes are considered potential drug targets due to their association with disease pathogenesis and the therapeutic potential of their encoded proteins. DGIdb provides comprehensive insights into drug-gene interactions and their clinical implications. We accessed DGIdb’s latest Interaction Data (updated December 2024) (Danishuddin et al., 2024). Finan et al. cataloged 4,479 druggable genes, offering a robust resource for target discovery (Finan et al., 2017). Integrating both datasets produced a more comprehensive list of druggable genes, several of which have been previously validated (Zhang et al., 2024).

### 2.3 Cis-eQTL datasets

The cis-eQTL data for whole blood were obtained from the eQTLGen consortium (https://eqtlgen.org/). Liver cis-eQTLs were taken from GTEx v10 (https://gtexportal.org). The eQTLGen consortium integrated 37 datasets comprising 25,482 whole blood samples from 31,684 participants, mostly of European descent (Võsa et al., 2021). The GTEx v.10 dataset included gene expression profiles from 54 tissues across 946 postmortem donors, primarily of European ancestry. These cis-eQTL data identify genetic variations affecting gene expression in blood and liver tissues. Variations are located within 1 Mb of each gene’s center and have minor allele frequencies >0.01 (Zhou et al., 2025).

### 2.4 PBC GWAS dataset

The GWAS summary data for PBC included 16,489 European controls and 8,021 European patients, covering 5,004,018 SNPs (GWAS ID: ebi-a-GCST90061440) (Cordell et al., 2021). Sourced from the latest and largest PBC GWAS (Wu et al., 2024), this dataset was retrieved as a Variant Call Format file via the IEU Open GWAS project (https://gwas.mrcieu.ac.uk/).

### 2.5 Mendelian randomization analysis

GWAS have identified numerous disease-associated risk SNPs. However, GWAS findings cannot directly translate to drug discovery for two reasons: they only identify associative signals, and SNPs themselves do not encode proteins (though they may influence gene expression). MR leverages exposure-associated SNPs as IVs to infer causal relationships between exposures and outcomes (Li X. et al., 2023). MR analysis requires three key assumptions: (Patel et al., 2021): genetic variants must be strongly associated with the exposure; (Geng et al., 2025); no association with confounding variables; and (Selmi et al., 2011) any effect on the outcome must be mediated solely through the exposure (Davies et al., 2018). In this study, MR analysis was performed using the TwoSampleMR R package (v0.6.8). Pharmacogenomic cis-eQTLs served as exposure data, with PBC as the outcome. IVs were constructed by filtering SNPs with p < 5 × 10^−8^, followed by linkage disequilibrium (LD) pruning (r^2^ < 0.001within a 10,000 kb window) using 1000 Genomes Project European samples. Weak IVs were identified via F-statistic calculation (F = (β/SE)^2^, where β is the SNP-exposure regression coefficient and SE is its standard error), excluding SNPs with F < 10 to minimize bias (Yang et al., 2025; Sun X. et al., 2024). Post-data integration, MR analysis was performed on the selected SNPs. The Wald ratio method was utilized when only one SNP was available for analysis. In cases with multiple SNPs, the inverse-variance weighted (IVW) method with random effects was employed (Zhang et al., 2024). The heterogeneity of individual causal effects among SNPs was assessed using Cochran’s Q test, and MR Egger intercept was employed to evaluate SNP pleiotropy (Tong et al., 2025). Statistical significance was defined as p < 0.05.

### 2.6 Colocalization analysis

SNPs can sometimes lie within regions of two or more genes, and their impact on disease (here referring to PBC) may be influenced by the interplay of different genetic factors. To evaluate whether multiple genetic associations correspond to the same causal variation at the same genomic location, we employed colocalization analysis to identify potential shared causal genetic variation between PBC and cis-eQTLs. Specifically, for the significant MR results identified, we conducted colocalization analysis using the R package “coloc” (version 5.2.3) on SNPs within ±1 Mb of the transcription start site (TSS) for each gene associated with PBC risk and cis-eQTLs. The probability of a SNP being associated with PBC is denoted as P1, the probability of being a significant cis-eQTL as P2, and the probability of being associated with both PBC and cis-eQTL as P12. All probabilities were set to default values (P1 = 1 × 10^−4^, P2 = 1 × 10^−4^, and P12 = 1 × 10^−5^). We used posterior probabilities (PP) to measure the support for all hypotheses, labeled as PPH0 to PPH4: PPH0 indicates no association with any traits; PPH1 indicates association with gene expression but no correlation with PBC risk; PPH2 indicates association with PBC but no relation to gene expression; PPH3 indicates correlation with both PBC risk and gene expression, suggesting a significant causal relationship; PPH4 indicates correlation with both PBC risk and gene expression, indicating a shared causal relationship. In light of the limitations of colocalization analysis, and to enhance the sensitivity for detecting colocalization signals while expanding the range of potential drug targets, we have constrained subsequent analyses to genes with PPH4 values ≥0.5 (You et al., 2025; Ke et al., 2025).

### 2.7 Phenome-wide association analysis

To establish causal links between identified druggable genes and disease traits, and to evaluate potential side effects or alternative indications, we performed a Mendelian randomization phenome-wide association study (MR-PheWAS) analysis. This analysis used Finnish population-based outcome phenotypes from the FinnGen database, specifically Release 11, which is accessible at https://r11.finngen.fi/.

### 2.8 Enrichment analysis

To investigate the functional traits and biological importance of the pre-selected potential druggable genes, Gene Ontology (GO) enrichment analysis and Kyoto Encyclopedia of Genes and Genomes (KEGG) pathway analysis were performed using the R package “clusterProfiler” (version 4.12.6) (Yang et al., 2025). GO analysis, encompassing biological process (BP), molecular function (MF), and cellular component (CC) terms, aimed to determine gene activity patterns, functional roles, and cellular localization. KEGG pathway analysis provided insights into metabolic pathways, crucial for understanding biological functions and potential drug action mechanisms. These analyses systematically characterized the functional networks of the genes, establishing a biological basis for drug development.

### 2.9 Protein–protein interaction network construction

The protein-protein interaction (PPI) network visually presents interaction relationships among key druggable genes. We generated this network via STRING (https://string-db.org/) by setting the minimum interaction score at 0.15 and keeping other parameters at default (Szklarczyk et al., 2023).

### 2.10 Candidate drug prediction and molecular docking

The Drug Signatures Database (DSigDB, https://dsigdb.tanlab.org/DSigDBv1.0/) contains 22,527 gene sets and 17,389 compounds linked to 19,531 genes (Li et al., 2024; Xie et al., 2025). We queried DSigDB with previously identified druggable genes to predict candidate drugs and their target activities. To further assess the practical feasibility of candidate drugs, we performed molecular docking simulations for candidate drugs and their corresponding target proteins to evaluate the binding affinity and interaction patterns between drugs and their targets, identifying high-affinity ligands for subsequent experimental validation. Drug structure data were obtained from PubChem (https://pubchem.ncbi.nlm.nih.gov/) in SDF format. Preparation involved generating major protonated/tautomeric forms (pH 7.4), followed by energy minimization (MMFF94), hydrogen addition, Gasteiger charge assignment, and conversion to PDBQT format. Protein structures were retrieved from the PDB (https://www.rcsb.org/). Preparation included removal of crystallographic water, irrelevant ions, and heteroatoms, followed by polar hydrogen addition (pH 7.4), residue protonation correction, and Gasteiger charge assignment (Chen et al., 2025). Molecular docking of candidate drugs with their protein targets was conducted using AutoDock Vina 1.2.2, and results were visualized in 3D with PyMOL (Eberhardt et al., 2021).

### 2.11 Animal experimental validation

#### 2.11.1 Chemicals and reagents

Galangin (GAL, purity >98%) was purchased from Shanghai Yuanye Biotechnology Co., Ltd. (Shanghai, China). α-Naphthyl isothiocyanate (ANIT, purity >98%) and ursodeoxycholic acid (UDCA, purity ≥99%) were obtained from Shanghai Aladdin Biochemical Technology Co., Ltd. (Shanghai, China). Assay kits for alanine aminotransferase (ALT), aspartate aminotransferase (AST), total bile acids (TBA), total bilirubin (TBIL), direct bilirubin (DBIL), alkaline phosphatase (ALP), and γ-glutamyl transpeptidase (γ-GT) were purchased from Nanjing Jiancheng Bioengineering Institute (Jiangsu, China). Enzyme-linked immunosorbent assay (ELISA) kits for interleukin-1β (IL-1β), interleukin-6 (IL-6), and tumor necrosis factor-α (TNF-α) were purchased from 4A Biotech Co., Ltd. (Jiangsu, China). All other experimental consumables were commercially procured.

#### 2.11.2 Mouse grouping, model establishment, and sample collection

**FIGURE 2:**
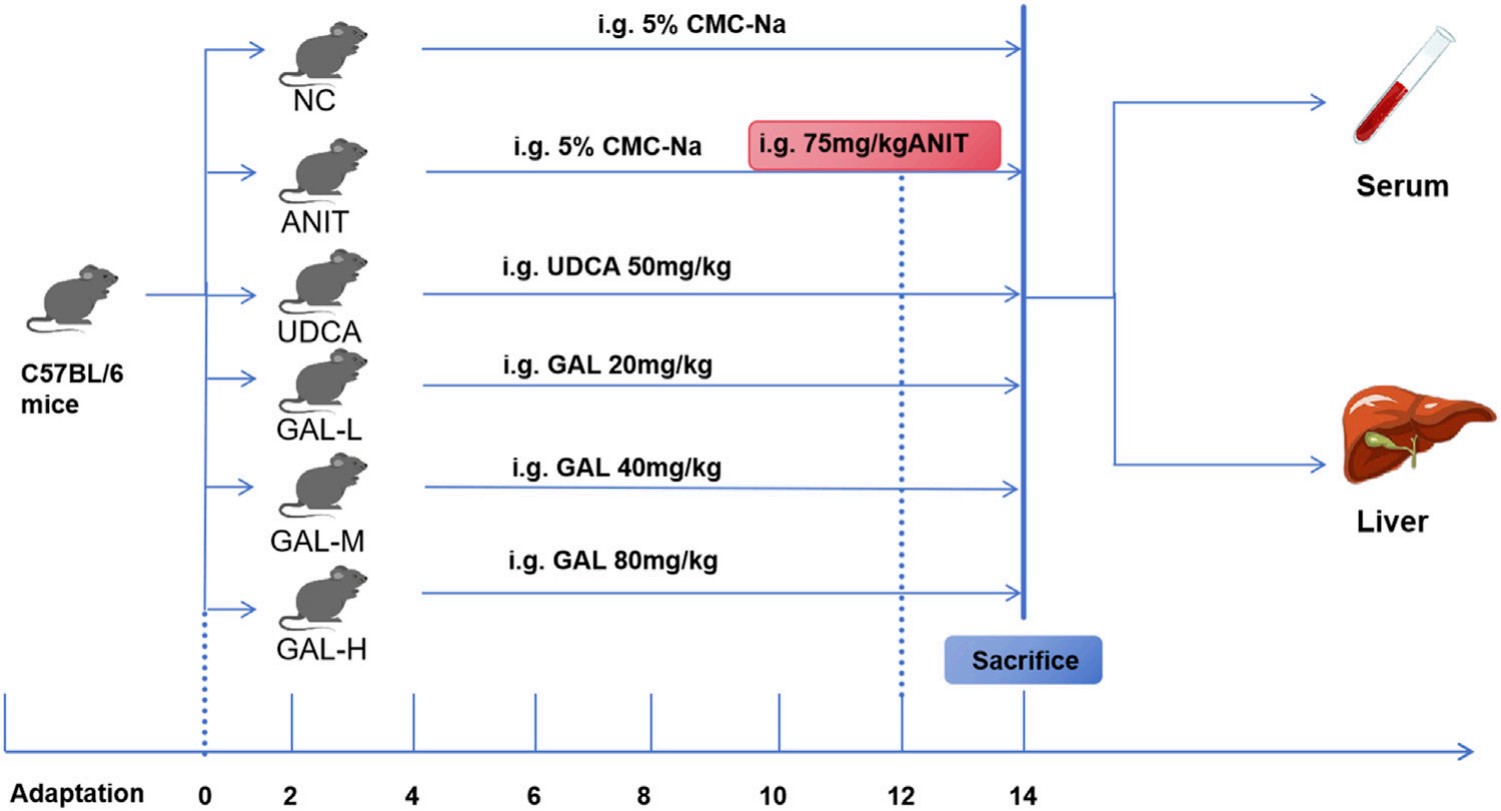
Flowchart of the GAL experiment for treating PBC mice.

Six-week-old female C57BL/6 mice were purchased from Henan Sike Beisi Biotechnology Co., Ltd. (Anyang, China). The mice were housed in a controlled environment with a temperature range of 20 °C–24 °C and relative humidity of 60 ± 5%, following a 12-h light/dark cycle, and were provided with food and water *ad libitum*. All animal experiments were approved by the Ethics Committee of the Hebei Medical University Third Hospital (Ethics No. Z2025-027-01) and performed in accordance with institutional guidelines for animal care and use. After 1 week of acclimatization, the mice were randomly divided into 6 groups by using a random number table, with six mice per group (n = 6): NC (negative control), ANIT (α-naphthylisothiocyanate), UDCA (ursodeoxycholic acid), GAL-L (low-dose galangin), GAL-M (medium-dose galangin), and GAL-H (high-dose galangin). The NC and ANIT groups received 0.5% sodium carboxymethyl cellulose (CMC-Na) solution (vehicle) by gavage, while the UDCA and GAL groups received their respective drugs preventively by gavage. The UDCA group received ursodeoxycholic acid at 50 mg kg^-1^, and the GAL-L, GAL-M, and GAL-H groups received GAL at 20, 40, and 80 mg kg^-1^ respectively, with daily treatments for 12 days. On day 12, 2 hours after the last treatment, the NC group received olive oil by gavage, and the other groups received a mixture of 75 mg kg^-1^ ANIT with olive oil. After ANIT treatment, groups continued with their original gavage schedule for two additional days. The setup of the ANIT-induced mouse model and the doses of GAL and ursodeoxycholic acid were based on previous studies (Sun L. et al., 2024; Scarlata et al., 2025; Tan et al., 2021; Zhou et al., 2022; Su et al., 2021). The experimental design is outlined in Figure 2. At termination, mice were euthanized, and blood and liver samples were collected.

#### 2.11.3 Histological examination

Liver tissue samples from mice were fixed with 4% paraformaldehyde, embedded in paraffin, and sectioned. After deparaffinization, sections were stained with hematoxylin-eosin (H&E). Histopathological changes, including portal tract inflammation and bile duct hyperplasia, were blindly evaluated by pathologists unaware of the study design using optical microscopy.

#### 2.11.4 Analysis of serum biochemical indicators

Serum samples from mice were collected, and the levels of ALT, AST, TBA, TBIL, DBIL, ALP, and γ-GT were measured using commercial test kits (Nanjing Jiancheng, Jiangsu, China), according to the manufacturer’s instructions.

#### 2.11.5 Detection of inflammatory cytokine levels in liver tissue

Liver tissues from mice were collected, and levels of IL-1β, IL-6, and TNF-α in the homogenates were measured using ELISA kits (4A Biotech, Jiangsu, China), according to the manufacturer’s instructions.

### 2.12 Statistical analysis

Statistical analyses were performed using IBM SPSS Statistics 29 and GraphPad Prism 10.1.2 software. Data are presented as the mean ± standard deviation (SD). Statistical significance was assessed by one-way analysis of variance (ANOVA), followed by Tukey’s *post hoc* test for multiple comparisons between groups. A p-value of less than 0.05 was considered to indicate statistical significance.

## 3 Results

### 3.1 Statistical analysis of the druggable genome

Using data from DGIdb v5.0.8, we identified 5,012 genes with potential druggable properties (Supplementary Table S1). Another 4,463 druggable genes were sourced from a prior review (Supplementary Table S2). Upon data integration, 6,888 unique genes bearing Human Genome Organization naming committee designations were obtained for subsequent analysis (Supplementary Table S3).

### 3.2 Candidate drug genes

By intersecting cis-eQTLs in blood and liver tissues with druggable genes, we identified druggable cis-eQTLs. The blood eQTL dataset included 4,439 gene symbols, whereas the liver tissue eQTL dataset comprised 1,669 gene symbols. For cis-eQTLs in blood tissue, the number of SNPs initially screened using the criterion of p < 5 × 10^−8^ ranged from 1 to 20,388 (mean = 566); after LD pruning, this number ranged from 1 to 35 (mean = 5), with an average F-statistic of 217; for cis-eQTLs in liver tissue, the number of SNPs after initial screening (using the same p < 5 × 10^−8^ criterion) ranged from 1 to 3,140 (mean = 98), and following LD pruning, the number of SNPs ranged from 1 to 6 (mean = 1) with an average F-statistic of 110. Using the Wald ratio or IVW method for MR analysis, we identified 183 significant genes associated with PBC in blood and 32 in liver tissue. Notably, RPS6KL1 and SP1 were significant in both tissues: blood (RPS6KL1 OR = 1.23, SP1 OR = 0.84) and liver (RPS6KL1 OR = 1.13, SP1 OR = 0.81). Complete MR results are shown in Supplementary Tables S4, S5. MR-Egger regression detected pleiotropy for HLA-DRB6 (P = 0.048) and QDPR (P = 0.039) (Supplementary Table S6. Additionally, the Cochran Q test indicated heterogeneity for HLA-DRB6 (P = 5.90 × 10^−5^), HLA-G (P = 0.010), MICB (P = 0.035), and TUBB (P = 0.015) (Supplementary Table S7).

### 3.3 Colocalization analysis

**FIGURE 3:**
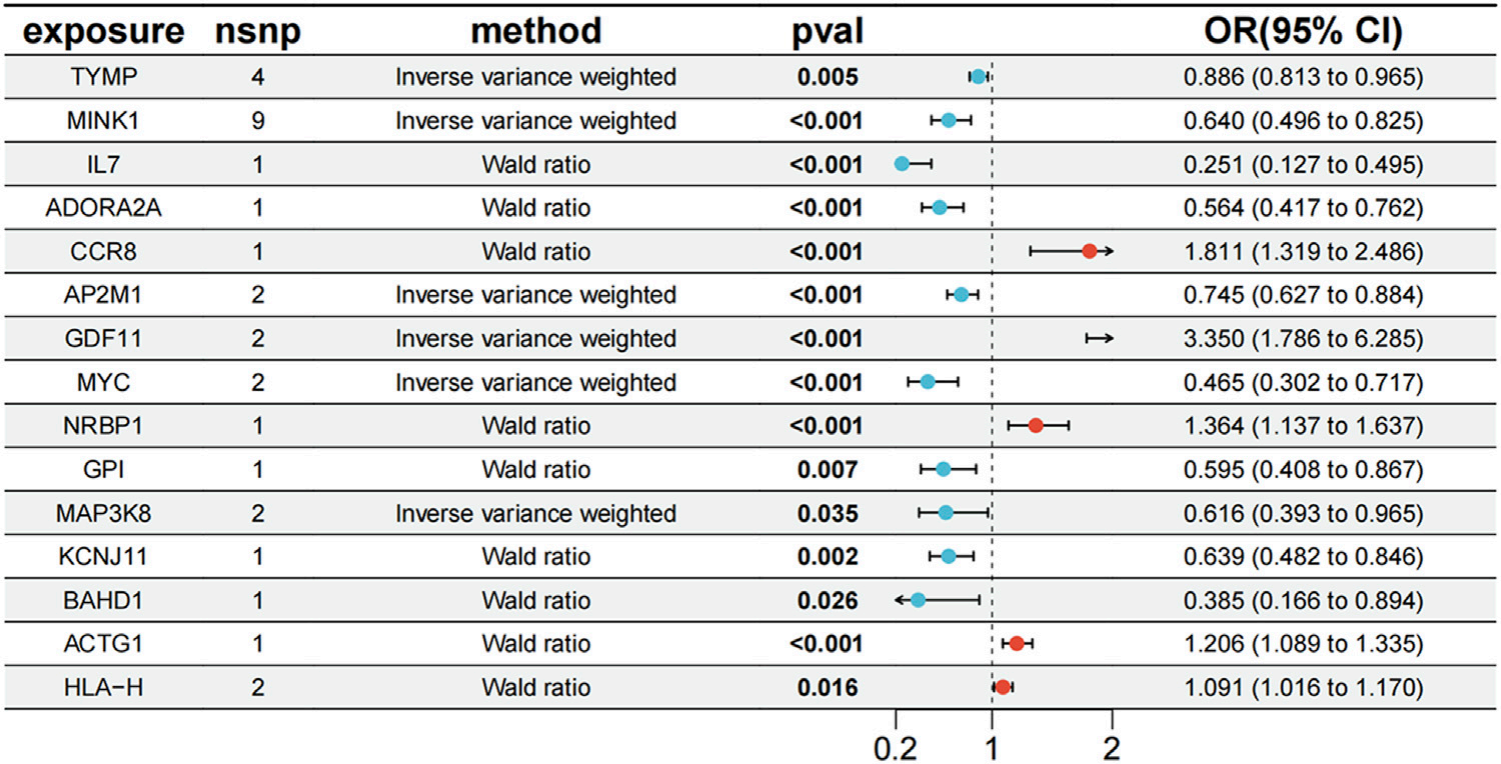
Forest plot of 15 significant genes associated with PBC.

To evaluate the potential shared causal variants between cis-eQTLs and PBC outcomes, we performed colocalization analysis on the druggable target genes that yielded significant MR results. The results showed that among the 178 significant genes previously identified in blood, 13 genes had a PPH4 value >0.5, specifically: TYMP, MINK1, IL7, ADORA2A, CCR8, AP2M1, GDF11, MYC, NRBP1, GPI, MAP3K8, KCNJ11, and BAHD1. Additionally, among the 32 significant genes in liver tissue, 2 genes (ACTG1 and HLA-H) had a PPH4 value >0.5 (Supplementary Table S8). Although RPS6KL1 and SP1 were associated with PBC in both tissues, they were excluded post-analysis. Genes with an odds ratio (OR) < 1 were protective, suggesting reduced disease risk, while those with an OR > 1 were risk-associated, indicating increased susceptibility (Xie et al., 2025). Among the 15 evaluated genes, TYMP (OR = 0.886, 95% CI [0.813, 0.965]), MINK1 (OR = 0.640, 95% CI [0.496, 0.825]), IL7 (OR = 0.251, 95% CI [0.127, 0.495]), AP2M1 (OR = 0.745, 95% CI [0.627, 0.884]), MYC (OR = 0.465, 95% CI [0.302, 0.717]), KCNJ11 (OR = 0.639, 95% CI [0.482, 0.846]), BAHD1 (OR = 0.385, 95% CI [0.166, 0.894]), ADORA2A (OR = 0.564, 95% CI [0.417, 0.762]), GPI (OR = 0.595, 95% CI [0.408, 0.867]), and MAP3K8 (OR = 0.616, 95% CI [0.393, 0.965]) were potential protective genes. Conversely, GDF11 (OR = 3.350, 95% CI [1.786, 6.285]), CCR8 (OR = 1.811, 95% CI [1.319, 2.486]), HLA- H (OR = 1.091, 95% CI [1.016, 1.170]), NRBP1 (OR = 1.364, 95% CI [1.137, 1.637]), and ACTG1 (OR = 1.206, 95% CI [1.089, 1.335]) were likely PBC risk genes (Figure 3).

### 3.4 Phenome-wide association study results

In this study, we leveraged the latest 11th-release GWAS data from the FinnGen database to perform PheWAS analyses on significant genes identified via integrated analysis. Results showed that these target genes associate with multiple diseases or phenotypes. After correcting for multiple testing using the false discovery rate (FDR), we identified a significant association between the GPI gene and corneal scarring. The TYMP gene exhibited strong associations with several conditions, including other enteritis, intestinal obstruction, shoulder joint disorders, chronic lymphocytic leukemia, non-infectious enteritis, and intestinal stenosis. Manhattan plots of MR-PheWAS results for GPI and TYMP are shown in Supplementary Figure S1.

### 3.5 Enrichment analysis

**FIGURE 4:**
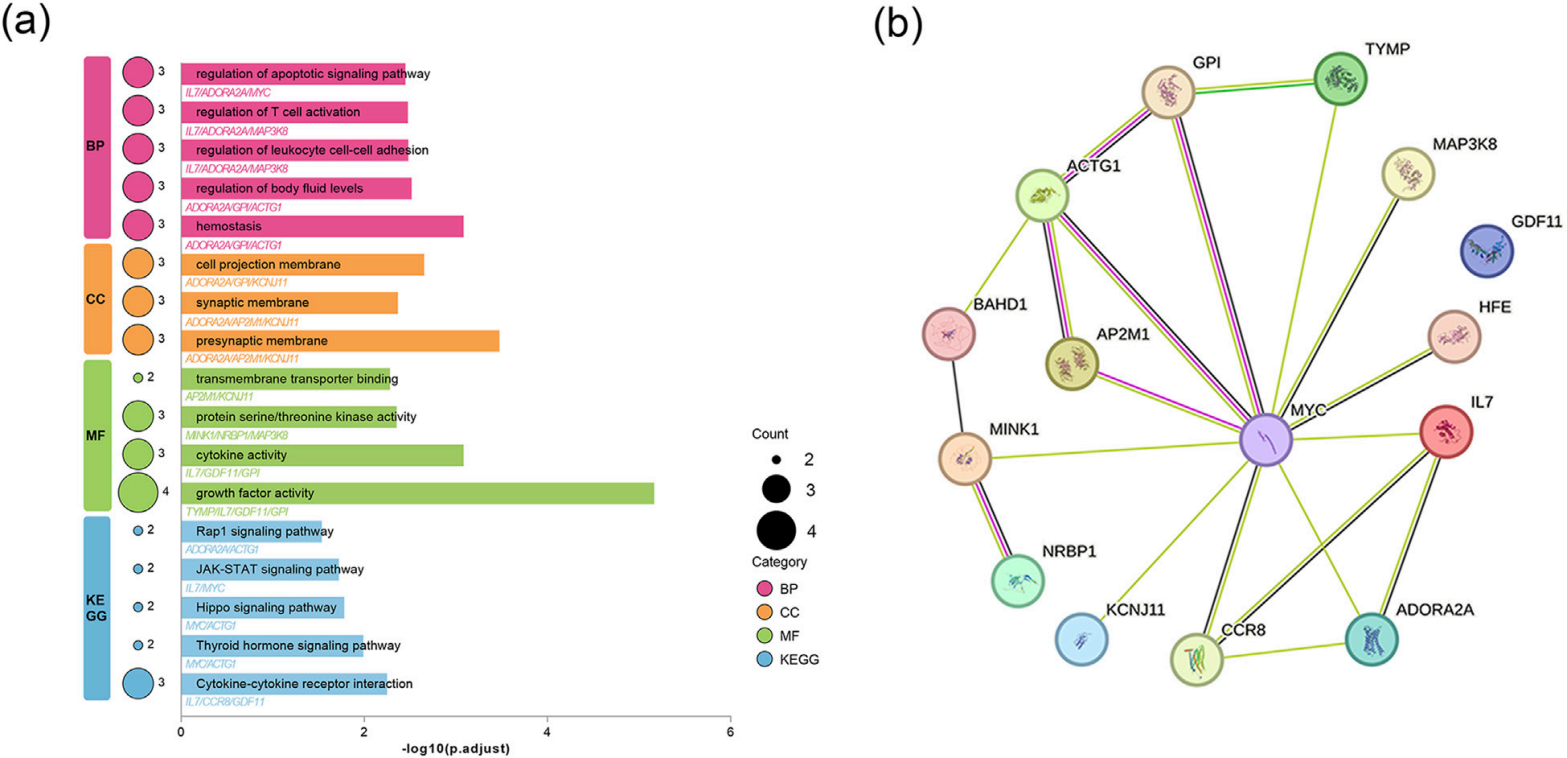
Enrichment analysis and construction of PPI network for candidate genes. **(a)** GO enrichment results for three terms and KEGG enrichment results. **(b)** PPI network built with STRING.

Through GO analysis of 15 potential targets, we found that these targets are significantly enriched in biological processes such as regulation of apoptotic signaling pathway, regulation of T cell activation, regulation of leukocyte cell-cell adhesion, and regulation of body fluid levels, which are closely related to immune responses, inflammation, and apoptosis. Cellular component analysis indicated that these target genes are primarily enriched in the cell projection membrane, synaptic membrane, and presynaptic membrane, reflecting their importance in intercellular signal transmission. In terms of molecular function, there is a high level of enrichment in growth factor activity, along with cytokine activity and protein serine/threonine kinase activity. KEGG analysis showed that the target genes are enriched in several key pathways, including cytokine-cytokine receptor interaction, thyroid hormone signaling pathway, Hippo signaling pathway, JAK-STAT signaling pathway, and Rap1 signaling pathway. These pathways significantly influence immune-inflammatory responses and cholangiocyte proliferation. Overall, these findings enhance our understanding of how targets regulate immune homeostasis, inflammatory signaling, cell apoptosis, and related biological processes (Figure 4a).

### 3.6 Protein–protein interaction network construction

We loaded 15 drug target genes into the STRING database to construct a PPI network. As shown in Figure 4b, the results identified a protein interaction pathway with 14 nodes and 13 edges. Notably, MYC, GPI, and ADORA2A showed higher connectivity within this network (Figure 4b).

### 3.7 Candidate drug prediction and molecular docking

**FIGURE 5:**
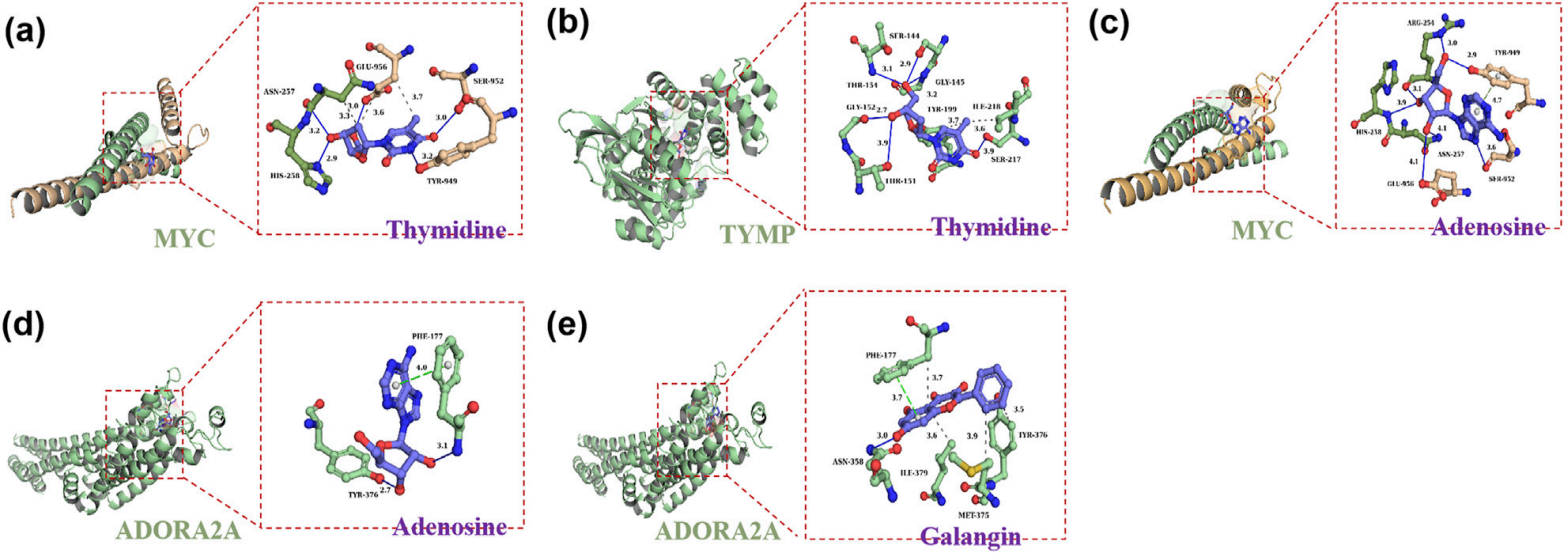
Molecular docking results of available proteins and drugs. **(a)** Docking of MYC with thymidine; **(b)** Docking of TYMP with thymidine; **(c)** Docking of MYC with adenosine; **(d)** Docking of ADORA2A with adenosine; **(e)** Docking of ADORA2A with galangin.

In this study, DSigDB was employed to predict potential effective interventional drugs, with the screening criteria set as p < 0.05 and a comprehensive score >500 (Raulo and Qazi, 2024). Based on p-values, the top 10 potential interventional drugs were listed (Table 1). Analysis revealed that selenium (CTD 00006731), thymidine (CTD 00006888), adenosine (CTD 00005319), GAL (CTD 00008147), and chlorpropamide (CTD 00005649) ranked among the top five, emerging as the most critical candidates. Specifically, selenium was associated with eight genes: GPI, ADORA2A, MYC, MINK1, MAP3K8, AP2M1, TYMP, and ACTG1. Thymidine was linked to two genes (MYC and TYMP), adenosine to ADORA2A and MYC, GAL to ADORA2A, and chlorpropamide to KCNJ11. Previous studies have confirmed that selenium, while capable of alleviating cholestasis by reducing oxidative stress and inflammatory responses, carries potential toxic side effects and thus poses a risk of selenium poisoning (Nassef et al., 2021; Vinceti et al., 2018; Hadrup and Ravn-Haren, 2023). In contrast, chlorpropamide may induce cholestatic liver injury (Reichel et al., 1960; Schneider et al., 1984). The effects of adenosine, thymidine, and GAL on PBC are currently unexplored. To further assess the feasibility of candidate drugs, molecular docking technology was used in subsequent research to analyze the binding affinity between candidates and their corresponding targets, thereby evaluating the druggability of target proteins. The AutoDock Vina 1.2.2 docking software was utilized to analyze the binding sites and interactions between adenosine, thymidine, GAL and the proteins encoded by their respective genes, with binding energies calculated for each interaction (results in Table 2; Figure 5). In the field of molecular docking research, a binding energy below −7 kcal/mol is generally considered to indicate strong ligand-receptor affinity, suggesting a favorable interaction from an energetic perspective (Cai et al., 2024). This study obtained three valid protein-drug docking results, among which ADORA2A and GAL exhibited the lowest binding energy (−8.8 kcal/mol), implying stable binding. Integrating drug prediction and molecular docking results, GAL was selected as the candidate drug for further in-depth research on potential therapeutic approaches for PBC.

**TABLE 1:** Candidate drug predicted by DSigDB.

Drug name	P-value	Combined score	Genes
Selenium CTD 00006731	0.0000	565.1631	GPI; ADORA2A; MYC; MINK1; MAP3K8; AP2M1; TYMP; ACTG1
Thymidine CTD 00006888	0.0001	2124.0450	MYC; TYMP
Adenosine CTD 00005319	0.0003	724.8138	ADORA2A; MYC
Galangin TTD 00008147	0.0082	685.0073	ADORA2A
Chlorpropamide CTD 00005649	0.0082	685.0073	KCNJ11
Tolbutamide CTD 00006903	0.0090	611.4626	KCNJ11
Trametinib CTD 00005130	0.0090	611.4626	MYC
Amifostine CTD 00005933	0.0097	551.0048	MYC
Glycerol CTD 00006038	0.0097	551.0048	MYC
Danazol TTD 00007428	0.0097	551.0048	MYC

**TABLE 2:** Molecular docking results of available proteins and drugs.

Target	PDB ID	Drug	PubChem ID	Binding energy (kcal/mol)
MYC	6G6J	Thymidine	5789	−5.2
TYMP	1UOU	Thymidine	5789	−7.6
MYC	6G6J	Adenosine	60961	−5.3
ADORA2A	5NM4	Adenosine	60961	−6.5
ADORA2A	5NM4	Galangin	5281616	−8.8

### 3.8 Animal experimental validation

#### 3.8.1 The effects of GAL on hepatic histopathology

**FIGURE 6:**
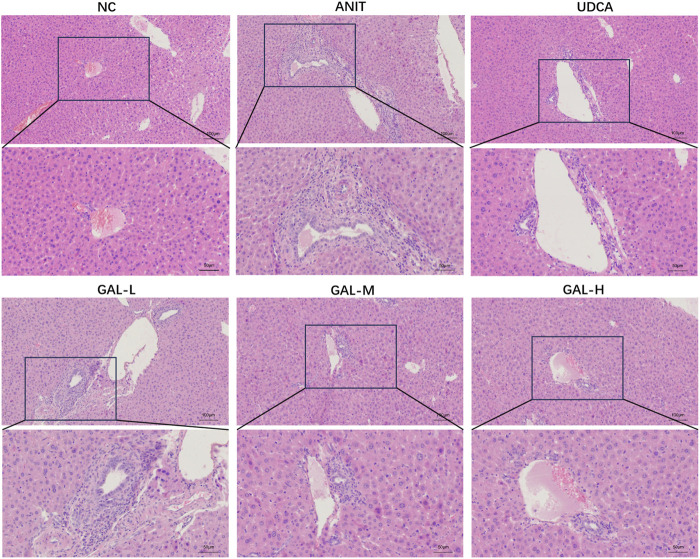
Effect of GAL on hepatic histopathological changes, as illustrated by hematoxylin-eosin (H&E) staining. GAL-L: GAL 20 mg kg^-1^ administered group; GAL-M: GAL 40 mg kg^-1^ administered group; GAL-H: GAL 80 mg kg^-1^ administered group. (Scale bars: 100 μm and 50 µm).

As shown in Figure 6, histological evaluation provided visual evidence for the protective effect of GAL against ANIT-induced intrahepatic cholestatic liver injury associated with PBC. Hepatic histopathological examination revealed that compared with the NC group, the ANIT group exhibited obvious histopathological changes caused by hepatic cholestasis, mainly manifested as significant inflammatory cell infiltration and bile duct hyperplasia around the small bile ducts in the portal area. In contrast, both the UDCA group and GAL groups showed improved inflammatory infiltration and bile duct hyperplasia, with more pronounced protective effects of GAL observed at medium and high doses.

#### 3.8.2 Effects of GAL on serum biochemical indicators

**FIGURE 7:**
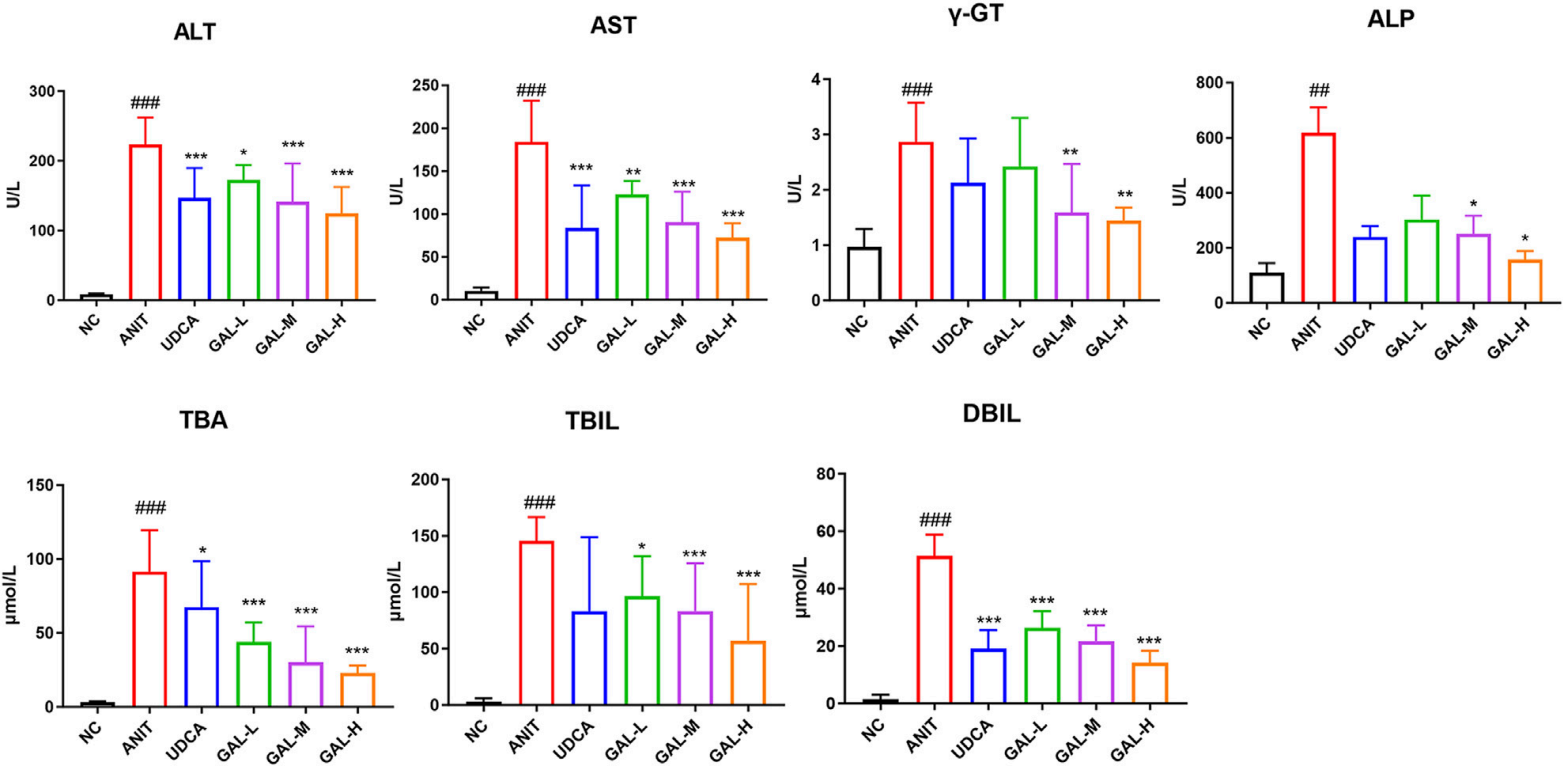
Effects of GAL on serum biochemical indicators related to liver function. Data are expressed as mean ± SD and were analyzed using one-way ANOVA. ##p < 0.01, ###p < 0.001 vs. the NC (negative control) group; ***p < 0.001, **p < 0.01, *p < 0.05 vs. the ANIT (model) group. GAL-L: GAL 20 mg kg^-1^ administered group; GAL-M: GAL 40 mg kg^-1^ administered group; GAL-H: GAL 80 mg kg^-1^ administered group.

Figure 7 shows the effects of GAL on the levels of ALT, AST, γ-GT, ALP, TBA, TBIL, and DBIL in the serum of mice. Compared with the NC group, the levels of these indicators in the ANIT group were significantly increased (###p < 0.001, ##p < 0.01), indicating the establishment of a PBC-related intrahepatic cholestasis model. The GAL treatment groups (GAL-L, GAL-M, GAL-H) and the positive control UDCA group showed the potential to improve liver function. Compared with the ANIT group, GAL and UDCA treatments reduced the levels of each indicator, among which the medium and high-dose GAL groups had more significant effects (***p < 0.001, **p < 0.01, *p < 0.05), suggesting that GAL may protect the liver by reducing liver injury and promoting bile secretion.

#### 3.8.3 Anti-inflammatory effects of GAL

**FIGURE 8:**
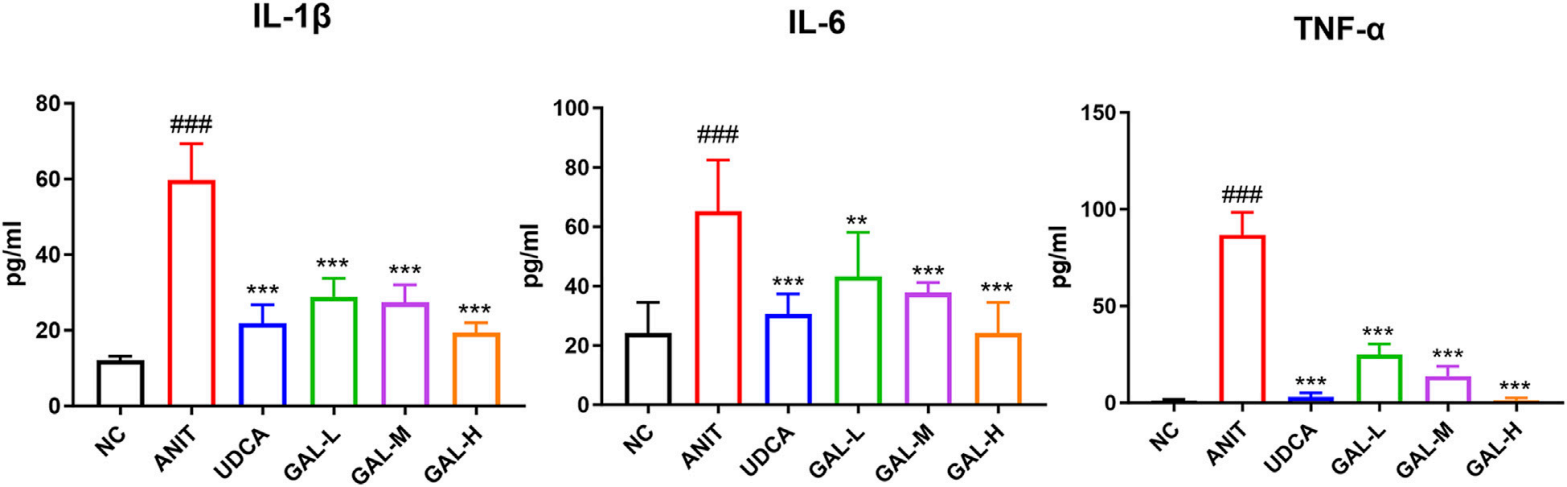
Effects of GAL on inflammatory cytokine levels in liver tissue related to liver inflammation. Data are expressed as mean ± SD and were analyzed using one-way ANOVA. ##p < 0.01, ###p < 0.001 vs. the NC (negative control) group; ***p < 0.001, **p < 0.01, *p < 0.05 vs. the ANIT (model) group. GAL-L: GAL 20 mg kg^-1^ administered group; GAL-M: GAL 40 mg kg^-1^ administered group; GAL-H: GAL 80 mg kg^-1^ administered group.

The Figure 8 shows GAL reduces inflammatory cytokines IL-1β, IL-6, and TNF-α in mouse serum. Compared to the NC group, ANIT group’s cytokines were significantly higher (###p < 0.001), showing a strong inflammatory response. GAL treatment groups and the positive control UDCA group had lower cytokine levels than the ANIT group, especially the medium and high-dose GAL groups (***p < 0.001, **p < 0.01, *p < 0.05). This suggests GAL may have anti-inflammatory properties and can alleviate liver inflammation.

## 4 Discussion

PBC is a chronic autoimmune cholestatic disease with incompletely understood pathogenesis and limited treatment options. Identifying effective therapies is crucial. MR analyses that integrate the druggable genome and cis-eQTL data have been used to find liver disease drug targets (Ma et al., 2025; Seagle et al., 2025). Building on this approach, we conducted a systematic druggable genome-wide MR analysis and identified 15 druggable genes significantly associated with PBC, including TYMP, MINK1, IL7, ADORA2A, CCR8, AP2M1, GDF11, MYC, NRBP1, GPI, MAP3K8, KCNJ11, BAHD1, ACTG1, and HLA-H. Subsequent PheWAS, enrichment analysis, and PPI network construction explored their pleiotropy, potential drug side effects, and biological significance. Finally, via drug prediction, molecular docking, and animal model validation, this study identified GAL as a therapeutic candidate for PBC, with its effects potentially mediated by the ADORA2A target. These findings provide novel therapeutic targets and drugs for PBC.

The 15 identified genes likely have distinct roles in PBC pathogenesis, although further validation is needed. For instance, ADORA2A, which encodes the Adenosine Receptor Subtype A2a (ADORA2A), critically modulates immunoinflammation by inhibiting the hepatic inflammatory cascade (Allard et al., 2023). Studies show that ADORA2A-deficient mice exhibit significantly elevated levels of pro-inflammatory factors in the liver (Zheng et al., 2022). Upon activation, ADORA2A also modulates molecules that inhibit oxidative stress and cell apoptosis (He et al., 2023; Ran et al., 2023). Furthermore, ADORA2A is expressed in various immune cells and can confer immunosuppressive effects by regulating the differentiation and function of macrophages, dendritic cells, and T cells (Ye et al., 2023; Nishikawa and Koyama, 2021). In addition to these functions, ADORA2A activation leads to G protein binding, initiating signaling pathways that elevate intracellular cyclic adenosine monophosphate levels. This subsequently activates protein kinase A (PKA) (Ye et al., 2023). As a serine/threonine kinase, PKA modulates the activity of various substrate proteins, including transcription factors, through phosphorylation. This pathway also influences cholesterol homeostasis by regulating the activity of the pregnane X receptor (Lichti-Kaiser et al., 2009). Moreover, ADORA2A indirectly modulates pathways such as phosphatidylinositol 3-kinase/protein kinase B (PI3K/AKT), which play crucial roles in autoimmune diseases and biliary disorders (Song et al., 2024; Ahmad et al., 2013; Wu et al., 2023). Therefore, ADORA2A may protect against PBC by mitigating inflammatory responses, suppressing oxidative stress and apoptosis, modulating immune functions, influencing bile acid metabolism, or engaging other related pathways. Other genes may also mediate PBC pathogenesis. TYMP, linked to apoptosis and inflammation, is downregulated in non-alcoholic fatty liver disease(NAFLD) and modulates the NF-κB pathway in acute liver injury models (Gautheron et al., 2022; Hou et al., 2022; Higashikawa et al., 2020), suggesting a protective role in PBC. MINK1 mitigates autoimmune damage by inhibiting Th17 cell differentiation, and since PBC involves Th17-mediated injury (Fu et al., 2017), it may reduce biliary autoimmune attacks via suppressing Th17 activity. AP2M1 enhances autophagy, improves intestinal barrier function, and inhibits gut-associated lymphoid tissue activation, thereby reducing liver inflammation (Ganapathy et al., 2022; Hoffmanová et al., 2018). MYC (the first identified tumor-amplified proto-oncogene) participates in tumor processes and inhibits immune cells when overexpressed (Li J. et al., 2023). Given that excessive immune cell activation in PBC damages biliary epithelial cells, MYC may reduce biliary injury by inhibiting these immune cells. GPI regulates B cell tolerance and may control PBC progression by reducing autoreactive B cell activation (Satpute et al., 2008). BAHD1 inhibits the NF-κB pathway to decrease pro-inflammatory cytokines, and since sustained intrahepatic NF-κB activation in PBC drives massive pro-inflammatory factor release (Zhu et al., 2015), it may act via this pathway inhibition. CCR8 expression is closely associated with multiple immune diseases, and high expression is often linked to poor prognosis (Li et al., 2020), suggesting it may be a potential therapeutic target for PBC.

These genes are interconnected rather than isolated. PPI network analysis reveals interactions among their protein products, providing a systems-level perspective of PBC pathogenesis. Prior studies indicate that these genes may influence PBC pathogenesis through mechanisms such as immune regulation, biliary inflammation, and the gut-liver axis. GO and KEGG analyses also support this notion, showing enrichment in processes and pathways such as apoptosis regulation, T cell activation, cytokine-cytokine receptor interaction, JAK-STAT signaling pathway, and Rap1 signaling pathway. Many of these are closely linked to the regulation of cholangiocyte survival, apoptosis, and immune-inflammatory responses. This finding suggests that they may be involved in the development of PBC by affecting these key aspects. In summary, this study identified key PBC-associated genes via systematic druggable genome-wide MR analysis. Functional analysis indicates their enrichment in biological processes regulating immune homeostasis, inflammatory signaling, and apoptosis, among others. Given that these processes may play key roles in PBC pathogenesis, they might participate in PBC development by influencing these processes. However, cellular, animal, and clinical studies are needed to validate their specific functions, regulatory mechanisms, and clinical significance in PBC. Future research should focus on drug intervention studies targeting these genes and integrate multi-omics data to further confirm their functions, thus offering new insights for PBC treatment.

Drug prediction and molecular docking indicate that GAL binds strongly to ADORA2A, with low binding energy suggesting a stable interaction. This implies GAL may be a therapeutic candidate for PBC via ADORA2A. GAL has documented antioxidant, anti-inflammatory, anti-fibrotic, and anticancer activities and shows promise in liver conditions including drug-induced injury, NAFLD, alcoholic liver disease, hepatic fibrosis, and hepatocellular carcinoma (Zhang et al., 2020; Duan et al., 2024; Wang et al., 2013; Aladaileh et al., 2019). Some studies also report immunoregulatory effects relevant to autoimmune diseases (Tan et al., 2021). However, GAL’s role in cholestatic injury in PBC was previously unexamined. This study employed an ANIT-induced PBC mouse model, which can simulate the pathological features of intrahepatic bile duct inflammation and cholestasis (Tjandra et al., 2000; Chen et al., 2021; Mariotti et al., 2018). The results showed that GAL markedly reduced inflammatory cell infiltration around portal small bile ducts and bile duct hyperplasia. It also significantly lowered serum liver enzymes (ALT, AST, γ-GT, ALP, TBA, TBIL, DBIL) and hepatic inflammatory cytokines (IL-1β, IL-6, TNF-α). These findings fully support the therapeutic potential of GAL in alleviating liver injury in PBC. Consistent with GAL’s known hepatoprotective bioactivity, *in vivo* results confirm its anti-inflammatory properties, which help reduce portal tract inflammation and bile duct hyperplasia, thereby exerting both hepatoprotective and anti-cholestatic effects. Additionally, MR and colocalization analyses indicate a significant association, potentially causal, between ADORA2A and PBC risk. Molecular docking further corroborates a strong binding affinity between GAL and ADORA2A. Given the potentially diverse roles of ADORA2A in PBC pathophysiology, these findings suggest that GAL may effectively target ADORA2A as a treatment for PBC. Notably, GAL exhibits pharmacological activity by modulating multiple pathways. Research has shown that, besides its interactions with ADORA2A, GAL can alleviate inflammatory responses by inhibiting the NF-κB signaling pathway (Wang et al., 2021). It also activates the Nrf2 pathway, reducing oxidative stress, inflammation, and apoptosis, while modulating PPARγ expression to influence bile acid metabolism (Aladaileh et al., 2019; Chien et al., 2015; Li and Chiang, 2009). These multi-pathway regulatory properties not only elucidate GAL’s comprehensive advantages in alleviating liver injury, inflammation, and cholestasis induced by primary biliary cholangitis PBC, but also provide research directions for investigating the molecular mechanism network underlying its therapeutic effects on PBC, while suggesting future optimization of its application potential through multi-target synergistic regulation strategies. Given that off-target effects from target gene pleiotropy can compromise therapeutic safety, PheWAS results indicate associations between TYMP and intestinal diseases, and between GPI and corneal scars. Molecular docking identifies ADORA2A as the potential target of GAL, with no current evidence suggesting the aforementioned off-target effects. However, further studies are needed to systematically evaluate the *in vivo* binding specificity between GAL and ADORA2A, as well as the long-term safety of GAL. This will establish a foundation for GAL’s application in PBC treatment.

This study offers several strengths that bolster its scientific validity and translational significance. First, it leverages data-driven methods to identify novel therapeutic targets for PBC, addressing the high costs and lengthy timelines of traditional drug discovery. The integration of comprehensive drug-gene databases ensures the relevance of candidate genes to known drug targets, increasing the likelihood of successful drug repurposing or development. Second, the study strengthens causal inference by integrating cis-acting gene expression variations with PBC GWAS data through MR and colocalization analyses, effectively mitigating confounding and reverse causality. Furthermore, the multi-dimensional evidence provided by functional enrichment analysis, PPI analysis, and PheWAS supports the potential targets, covering functional characteristics, safety profiles, and therapeutic mechanisms. Adding to this evidence, drug prediction and molecular docking studies further highlight the therapeutic potential and target druggability. Critically, animal experiments confirming drug efficacy provide key preclinical support, significantly enhancing translational value. Specifically, the ANIT-induced model, by selectively injuring intrahepatic small bile ducts while sparing extrahepatic ducts, closely mimics the biliary phenotype of early-stage human PBC. This makes it a reliable model for pharmacodynamic validation (Chen et al., 2021). Finally, the choice of GAL, primarily derived from Alpinia galanga and propolis, adds another layer of translational advantage. As a natural flavonoid with a simple production process and low cost, GAL has a strong safety profile due to its long-term use in the food sector, suggesting its potential for rapid clinical translation and accelerating its journey from the lab to clinical settings (Priyono et al., 2024; Caruso et al., 2022).

Despite establishing a complete evidence chain from gene identification to drug validation, this study has several limitations. First, the GWAS and eQTL data used in this study are primarily derived from European populations, which limits their generalizability. Extending the research findings to individuals of other genetic ancestries requires further research and validation to ensure broader applicability. Second, while MR and colocalization analyses utilize large-scale population data, the absence of direct functional validation impedes definitive causal inference between genes and PBC. Third, the current research provides only a preliminary validation of the therapeutic efficacy of GAL. Although molecular docking experiments support the binding of GAL to ADORA2A, the precise molecular mechanism underlying this interaction remains unclear and requires further investigation. Finally, no single animal model can fully replicate all pathological features of human PBC at present; as a chemically induced injury model, the ANIT model cannot fully recapitulate the autoimmune characteristics of PBC. Future research will focus on validating the findings from the systematic druggable genome-wide MR analysis in multi-ethnic cohorts to enhance the generalizability of this study. Additionally, a quantitative analysis of ADORA2A expression levels in clinical samples will be conducted to establish its correlation with PBC phenotypes. Furthermore, optimization of animal models and incorporation of *in vitro* studies will investigate the protective effects of GAL on PBC comprehensively. Importantly, the molecular mechanisms underlying the interaction between GAL and ADORA2A will be investigated through structural biology approaches and mechanistic studies, as well as potential interactions with bile acid metabolism, immune cell infiltration, and other relevant pathways. This will deepen the understanding of GAL’s therapeutic potential and promote its development as a viable treatment option for PBC.

## 5 Conclusion

In conclusion, this study identified 15 druggable genes significantly associated with PBC via systematic druggable genome-wide MR analysis. Subsequently, the feasibility of GAL as a potential therapeutic agent was validated via drug prediction, molecular docking, and experiments using an ANIT-induced PBC mouse model. The therapeutic mechanism of GAL may be mediated by its action on ADORA2A. These findings provide new target directions and drug options for PBC treatment. Future research will focus on gene function verification, multi-model verification, and in-depth clarification of the molecular mechanism underlying the GAL-ADORA2A interaction, thereby laying a more solid and comprehensive theoretical and practical foundation for PBC treatment.

## Data Availability

The original contributions presented in the study are included in the article/Supplementary Material, further inquiries can be directed to the corresponding authors.

## Supplementary material

SUPPLEMENTARY FIGURE S1 Manhattan plots of the association between GPI and TYMP genes and disease phenotypes in MR-PheWAS; **(a)** MR-PheWAS Manhattan plot of GPI; **(b)** MR-PheWAS Manhattan plot of TYMP.

## Glossary

**PBC**: Primary biliary cholangitis
**MR**: Mendelian randomization
**eQTL**: Expression quantitative trait loci
**cis-eQTL**: cis-expression quantitative trait loci
**GWAS**: Genome-wide association study
**PheWAS**: Phenome-wide association study
**PPI**: Protein-protein interaction
**GAL**: Galangin
**ANIT**: α-Naphthylisothiocyanate
**UDCA**: Ursodeoxycholic acid
**LD**: linkage disequilibrium
**ALT**: Alanine aminotransferase
**AST**: Aspartate aminotransferase
**TBA**: Total bile acids
**TBIL**: Total bilirubin
**DBIL**: Direct bilirubin
**ALP**: Alkaline phosphatase
**γ-GT**: γ-Glutamyl transpeptidase
**IL-1β**: Interleukin-1β
**IL-6**: Interleukin-6
**TNF-α**: Tumor necrosis factor-α
**IL-2**: Interleukin-2
**H&E**: Hematoxylin-eosin
**DGIdb**: Drug-Gene Interaction Database
**IVs**: Instrumental variables
**IVW**: Inverse-variance weighted
**PP**: Posterior probabilities
**MR-PheWAS**: Mendelian randomization phenome-wide association study
**BP**: Biological process
MF: Molecular function
**CC**: Cellular component
**GO**: Gene Ontology
**KEGG**: Kyoto Encyclopedia of Genes and Genomes
**DSigDB**: Drug Signatures Database
**ELISA**: Enzyme-linked immunosorbent assay
**ANOVA**: Analysis of variance
**ADORA2A**: Adenosine Receptor Subtype A2a
**NAFLD**: non-alcoholic fatty liver disease
**PKA**: protein kinase A
