# Supplementary figures and images for: Identification of galangin as a therapeutic candidate for primary biliary cholangitis via systematic druggable genome-wide Mendelian randomization analysis and experimental validation
Source: Front Pharmacol. 2025 Oct 9;16:1674693. doi: 10.3389/fphar.2025.1674693 (PMC12545040; doi:10.3389/fphar.2025.1674693)

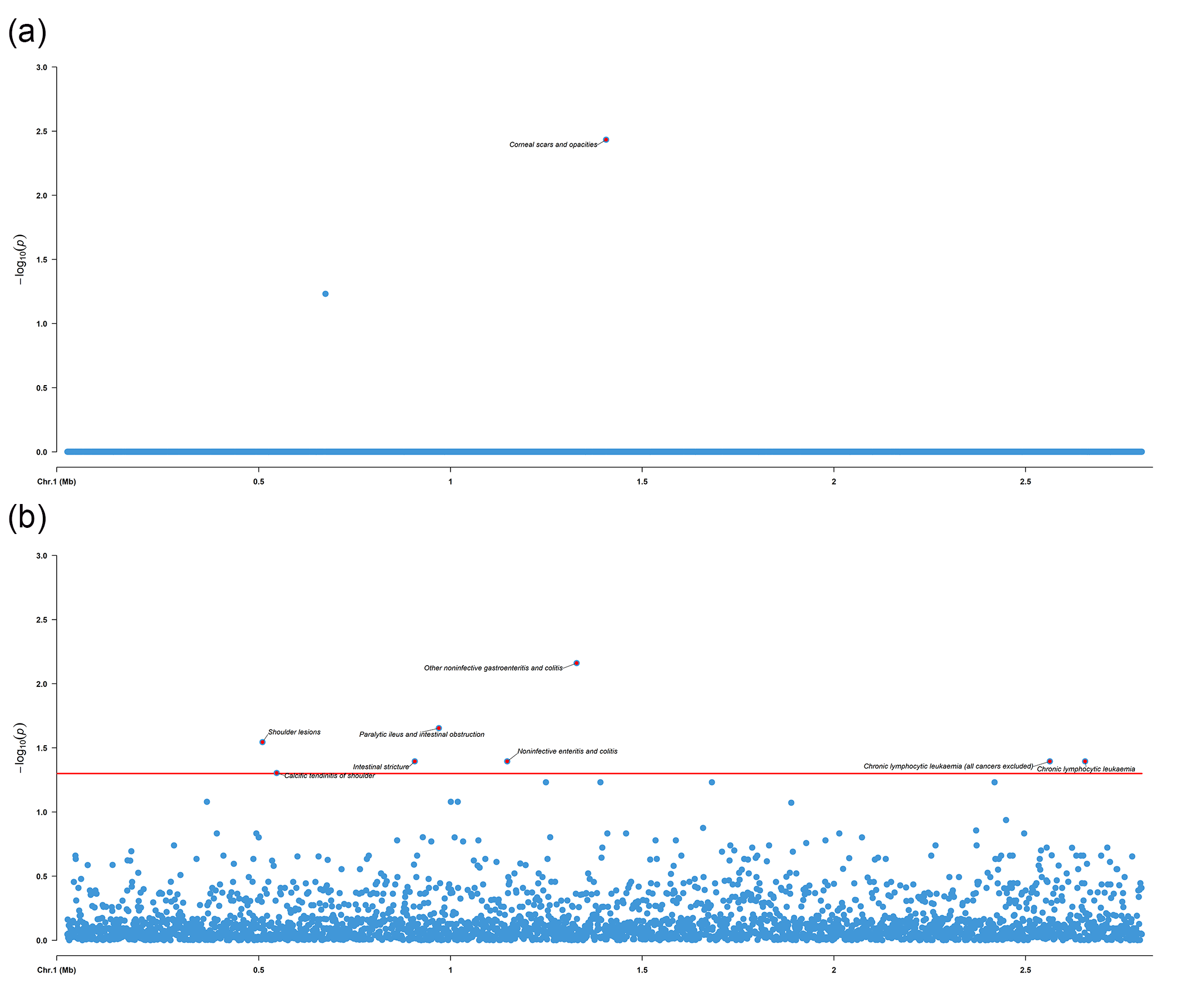

Supplement: Supplementary file 1 [file Image1.tif]
